# Influence Analysis of Polyvinyl Alcohol on the Degradation of Artificial Leather with Cellulose Nitrate Coating Originating from a Suitcase Stored in the Collection of the Auschwitz-Birkenau State Museum in Oświęcim, Poland

**DOI:** 10.3390/ma16217033

**Published:** 2023-11-03

**Authors:** Nel Jastrzębiowska, Anna Wawrzyk, Natalia Uroda

**Affiliations:** 1 Auschwitz-Birkenau State Museum, Więźniów Oświęcimia 20, 32-600 Oświęcim, Poland; 2 Silesian Park of Medical Technology Kardio-Med Silesia in Zabrze, M. Curie Skłodowskiej 10C, 41-800 Zabrze, Poland

**Keywords:** cellulose nitrate, degradation, polyvinyl alcohol, heritage, degradation

## Abstract

The aim of this study was to assess the influence of a protective layer of polyvinyl alcohol on the degradation process of artificial leather based on cellulose nitrate. Samples of the artificial leather were obtained from a suitcase dating back to the first half of the 20th century, not considered a historical artifact. The analysis involved Fourier-transform infrared spectroscopy with attenuated total reflection (FTIR-ATR) and X-ray photoelectron spectroscopy (XPS). Artificial aging was employed for the study. The artificial leather sample with a protective coating of polyvinyl alcohol on a cellulose nitrate base exhibited the lowest degree of degradation due to minimal chemical changes in cellulose esters. The obtained FTIR-ATR spectrum indicated significantly higher nitration of cellulose and, consequently, a lower degree of polymer degradation. The sample without the protective polyvinyl alcohol coating and the sample with the coating removed before artificial aging showed similar reactions.

## 1. Introduction

KL Auschwitz was the largest of the German Nazi concentration camps and extermination centers. During its period of operation, lasting over 4 years, at least 1,300,000 people from all over Europe were deported to the camp [1]. 

The Auschwitz-Birkenau State Museum (A-BSM) in Oświęcim takes care of items that remained from the victims of the Auschwitz concentration camp. Among these items are more than 3800 suitcases that were brought to the camp by deportees.

**Figure 1:**
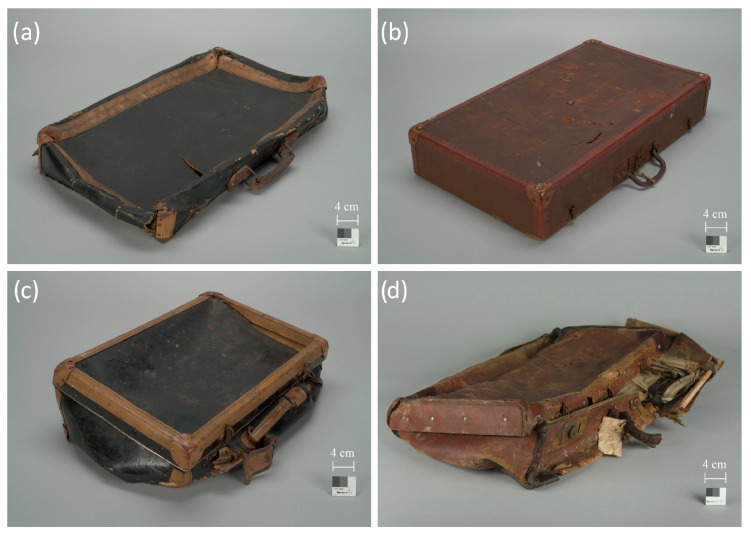
Suitcases covered with artificial leather, stored in the A-BSM Collections: (**a**) A suitcase covered with artificial leather based on cellulose nitrate, in black color, with the addition of dibutyl phthalate as a plasticizer, on a cotton substrate; (**b**) A suitcase covered with artificial leather based on cellulose nitrate, in brown color, with the addition of dibutyl phthalate, on a cotton substrate; (**c**) A suitcase covered with artificial leather based on cellulose nitrate, in black color, with the addition of dibutyl phthalate and rosin, on a paper substrate with a predominance of wood pulp; (**d**) A suitcase covered with artificial leather based on cellulose nitrate, in brown color, with the addition of dibutyl phthalate, on a cotton substrate. Photos by S. Mrozek.

Approximately 10% (about 400 pieces) of the suitcases in the collection are cardboard suitcases covered externally with artificial leather composed of a cellulose-nitrate-based coating applied to fabric or paper. In most cases, this material is coated in black, occasionally in various shades of brown (Figure 1).

Work on developing the method of applying cellulose nitrate coatings to fabric dates back to the 1850s [2]. Numerous processes were developed, products were patented, and formulas for producing waterproof compositions that imitated the appearance of natural leather were published. However, most of these did not progress beyond the experimental stage due to their limited flexibility, leading to utilization in narrow domains [3]. The term “artificial leather” was first used in a patent granted to W. Wilson and J. Storey in 1886. The recipe for the coating included amyl acetate, cellulose nitrate, castor oil, and pigment [4]. This composition was the first to compete with natural leather, possessing the required durability and flexibility. It became an inspiration for many patents involving similar mixtures using cellulose nitrate. These mixtures varied in the ingredient proportions and additives used [3,5]. The substrate material determined the strength properties of the resulting material, while the top coating influenced the leather-like appearance, resistance to abrasion and creasing, and water and air permeability. Materials imitating leather were most often prepared on substrates of densely woven cotton fabrics, with linen, wool, or silk also employed. Paper was also used as an underlying material [6]. For coatings on artificial leather, low-nitrogen cellulose nitrate (11–12% nitrogen) or celluloid (cellulose nitrate with camphor) was used. Initially, only celluloid was used, but this method had a significant drawback—a large amount of camphor was released on the surface of the product during drying [7]. Added solvents and diluents homogenized the cellulose nitrate with other components, giving the paste a viscous form, which was then evaporated from the covering layer. Cellulose nitrate, which formed brittle coatings, required the addition of a plasticizer. Initially, camphor was used for this purpose, but it was later replaced by castor oil, and then by tricresyl phosphate and tricresyl phosphate. From the 1820s, phthalates began to be used—dibutyl and dioctyl phthalates. Amyl and butyl phthalates and acetates were also used. Natural resins, such as rosin, copal, mastic, elemi, shellac, sandarac, and acaroid, as well as synthetic resins, including phenolic and phthalic ones, were introduced to improve the adhesion of the varnish to the substrate and enhance the gloss. Pigments and, later, synthetic dyes were added to the coating to provide opacity and color [3].

The suitcases that currently form part of the historical collections of A-BSM underwent a series of conservation treatments in the 1960s and 1970s. In the early 1960s, a Polish research institute specializing in fields related to the textile industry recommended the use of polyvinyl alcohol (PVA) for the protection of textiles to the first collection guardians of the Museum. During these procedures, one or two times, the entire surfaces of the suitcases were sprayed with a 10% solution of PVA. A layer of varnish formed on the surface of the suitcases, intended to provide protection against mechanical damage and chemical changes. Currently, PVA is found on A-BSM suitcases on materials such as vulcanized fiber, cardboard, paper, fabric, and artificial leather.

Since the mid-1990s, researchers and conservators in various centers around the world have proposed various methods to slow down degradation processes and improve aesthetics in plastic objects [8]. These suggestions include storage-related strategies [9,10,11], cleaning methods [10,12], consolidation [13], and filling of losses [13]. However, conservation methods for plastics, including cellulose nitrate, are still in the research, testing, and development stage [8]

Applying secondary coatings to cellulose nitrate objects was considered hazardous [14]. In the Auschwitz Museum, we encountered objects on which such coatings had been applied decades ago. Our studies aimed to scientifically assess the impact of applying a protective coating on cellulose nitrate in leather-like materials.

The literature does not provide information about the long-term effects of PVA on the surface of cellulose nitrate. Therefore, in this study, an attempt was made to evaluate the surfaces protected with this substance using highly specialized research techniques such as FTIR-ATR and XPS. The focus was mainly on analyzing chemical changes in the cellulose ester that indicate the progress of degradation [15,16].

The FTIR-ATR technique is commonly used to study surface changes in various fields of science. In the conservation of cultural heritage, this technique has been employed to investigate the degradation of surfaces made from cellulose and collagen [17,18]. This method has been applied to characterize nearly every material used in cultural heritage and its conservation [19,20,21], including cellulose nitrate [22,23,24,25]. It has also been used to assess the degradation process of cellulose nitrate [23,26,27,28]. It has been found to be suitable for evaluating molecular changes caused by degradation, particularly denitration and the formation of degradation products containing carbonyl groups. The FTIR-ATR technique has been used by many researchers to understand the degradation process of cellulose nitrate in historical artifacts [13,23,27,29]. During measurement, a small portion of the IR radiation reflected at the interface between the crystal and the sample penetrates into the sample, to a depth of 2–3 µm on average. Previous studies have shown that spectra obtained using this technique are useful for rapidly detecting chemical changes in such a material layer. A necessary condition for obtaining high-quality spectra is the close contact of the ATR crystal with the surface of the tested sample. This condition was met for all the analyzed materials. FTIR-ATR is an instrumental method based on analyzing the position and intensity of vibrations characteristic of different atomic groups present in a molecule. The position of a peak on the spectrum is related to a specific molecular structure or the presence of specific bonds or functional groups. Each shift in the position of a peak is directly related to a change in the angle, strength, or multiplicity of the chemical bond. Weakening or strengthening of the chemical bond shifts the characteristic band to lower or higher wavenumbers (frequencies). The absence of specific absorption bands indicates bond cleavage or rupture.

To understand the properties of cellulose nitrate in an even thinner near-surface layer of the material, the X-ray photoelectron spectroscopy (XPS) technique was employed. XPS is a highly advanced and specialized research method. By interacting in a high vacuum, radiation ejects photoelectrons from the core of atoms in the surface layer of the material being investigated. It is assumed that the penetration depth is 3–5 nm. The energy of the ejected electrons and their quantity depend on the atomic composition and chemical state of the surface. The type of chemical bonds between atoms, the degree of oxidation, and the neighboring atoms induce changes in the binding energy of electrons in the atoms. Recording photoelectrons in a hemispherical detector generates an XPS spectrum that provides qualitative and quantitative information about the chemical nature of material surfaces after appropriate processing and analysis. This technique is successfully employed to study historical surfaces [30]. The observation of changes in cellulose nitrate through XPS has been utilized in studies assessing the material’s suitability in industry as well [31,32]. However, it has not been used to test cellulose nitrate on historic objects.

To simulate processes that may occur on historical objects over the years, artificial aging is employed, involving elevated temperature, humidity, and light exposure. This method has been applied to evaluate degradation processes in cellulose nitrate in cultural artifacts [26,33,34] and is used in this study as well.

The aim of this study was to evaluate the impact of a polyvinyl alcohol layer on artificial leather based on cellulose nitrate. This research aimed to determine whether the hypothesis formulated back in the 1960s, suggesting that polyvinyl alcohol serves a protective function, is true, or whether polyvinyl alcohol leads to the retention of nitrogen compounds originating from degradation processes within the structure, potentially accelerating degradation due to the accumulation of catalyzing factors. Additionally, studies were conducted to assess whether the removal of polyvinyl alcohol by water would affect the degradation process of cellulose nitrate.

## 2. Materials and Methods

### 2.1. Study Objects

The samples for this study were obtained from a suitcase dating back to the early 20th century, not considered a historical artifact, stored in the collections of PMA-B. The presence of cellulose nitrate in the composition of the artificial leather’s coating was confirmed via Fourier-transform infrared attenuated total reflection spectroscopy (FTIR-ATR).

**Figure 2:**
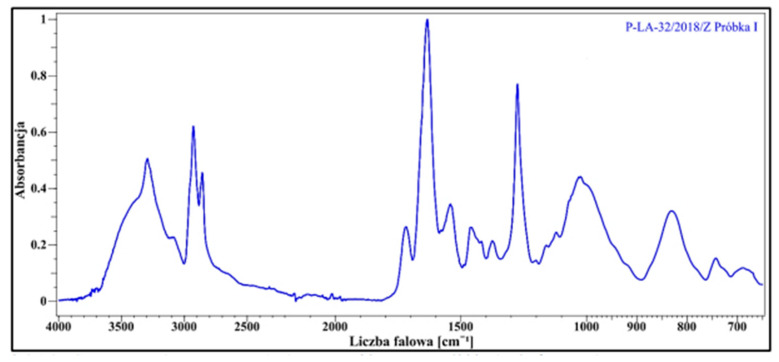
FTIR spectrum for a sample with confirmed presence of cellulose nitrate.

For this purpose, three samples from the suitcase were tested. The results for one of the samples confirmed the presence of cellulose nitrate, as shown in the FTIR spectrum below (Figure 2). The sample with detected cellulose nitrate was used for further tests and served as the starting material for three new samples subjected to the tests described in this publication.

**Figure 3:**
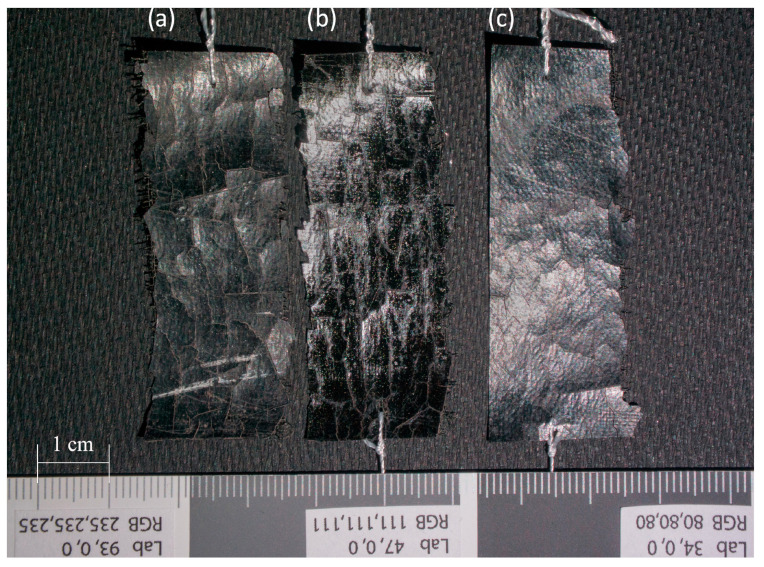
Samples of artificial leather with cellulose nitrate coating: (**a**) CN sample; (**b**) CN + PVA sample; (**c**) CN − PVA sample. Photo by M. Maciaszczyk.

**Figure 4:**
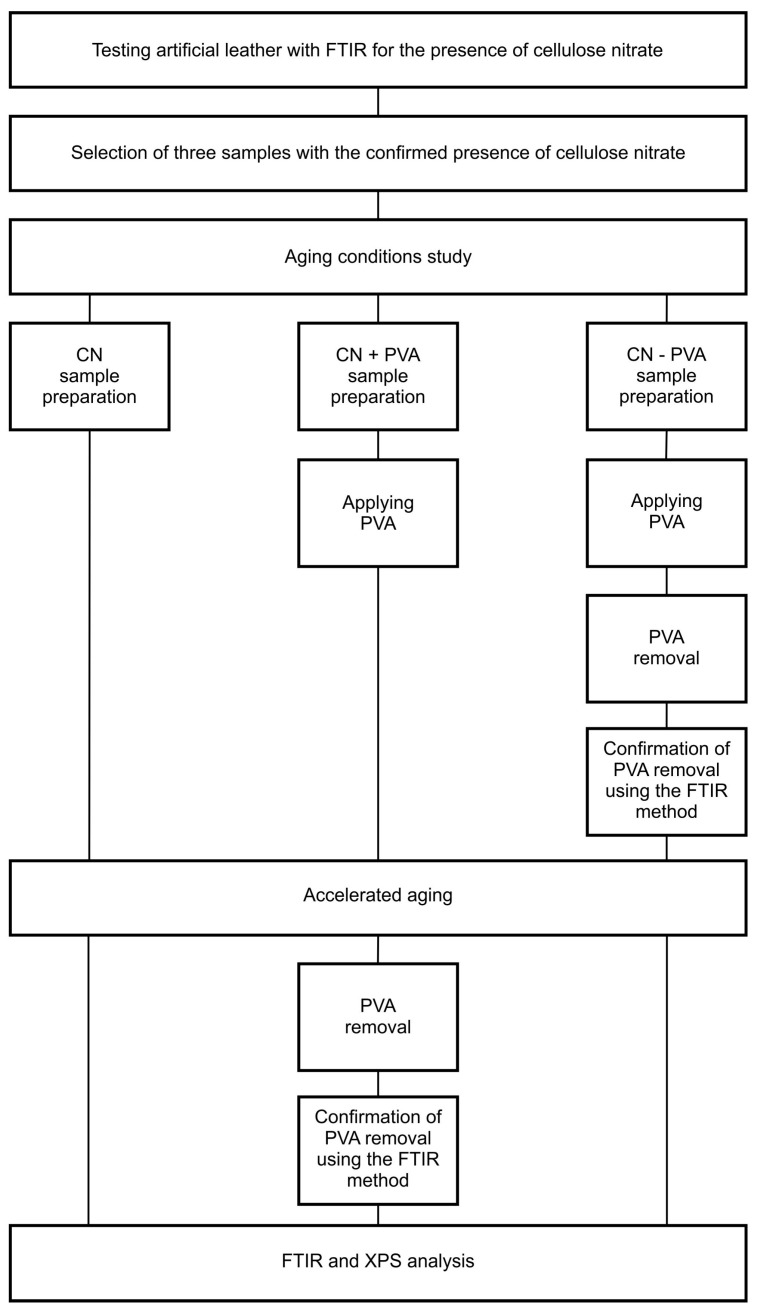
Experimental diagram.

Three samples were prepared (Figure 3 and Figure 4), each measuring approximately 5.4 cm × 2 cm: a sample of artificial leather without PVA coating (CN); a sample of artificial leather with one layer of PVA applied using a brush and then removed from the original material’s surface after artificial aging through hot water treatment (CN + PVA); and a sample of artificial leather with one layer of PVA applied using a brush, then removed by placing a cotton pad soaked in hot water for 5 min before artificial aging (CN − PVA). A 10% aqueous solution of polyvinyl alcohol (PVA) from Kremer in Poland was used to apply a coating with a thickness of 5 µm.

The CN and CN + PVA samples were prepared to determine the effect of the protective PVA coating on the degradation of cellulose nitrate in the artificial leather, whereas the CN PVA sample was prepared to investigate whether the removal of the secondary coating using water has a detrimental effect on cellulose nitrate.

### 2.2. Aging Conditions Study

Preliminary studies were conducted on artificial leather samples with an applied PVA coating to determine the optimal temperature (°C) and humidity (RH) conditions that would not result in the plasticization of the PVA. Four variations of climatic conditions were investigated: 70 °C and 60% RH, 60 °C and 50% RH, 50 °C and 50% RH, and 50 °C and 40% RH. Visual assessment by the conservators indicated that the PVA coating retained its appearance only under conditions of 40% RH and 50 °C. Thus, these conditions were chosen to age the prepared samples for 2186 h, i.e., 91 days.

The artificial aging process was conducted in an MKF240 climatic chamber from BINDER GmbH, Germany. During aging, UVA radiation in the range of 315–400 nm at 1.5 W was applied for a total duration of 1831 h, corresponding to 76 days.

### 2.3. Chemical Surface Structure Analysis of Cellulose Nitrate after Artificial Aging of Samples

#### 2.3.1. Fourier-Transform Infrared Attenuated Total Reflection Spectroscopy (FTIR-ATR)

To assess the influence of PVA on the chemical structure of the cellulose nitrate artificial leather’s surface, Fourier-transform infrared attenuated total reflection spectroscopy (FTIR-ATR) was utilized. ATR spectra of the surface layer of the examined samples were recorded using a diamond ATR accessory. Spectra were registered using a Thermo Nicolet 8700 FTIR spectrometer with Omnic 8.1 software, equipped with a Smart Orbit™ diamond ATR module and an MCT-A (Mercury Cadmium Telluride) detector from Thermo Scientific, Waltham, MA, USA. The high-sensitivity detector, cooled with liquid nitrogen, ensured signal stability in the mid-infrared spectral range of 4000–650 cm^−1^, independent of the scanning speed. ATR spectra underwent ATR correction, baseline correction, and scaled normalization, rendering these spectra equivalent to transmission spectra.

#### 2.3.2. X-ray Photoelectron Spectroscopy (XPS)

To evaluate changes in the elemental composition, types of functional groups, and chemical bonds on the surface of the tested samples, high-energy X-ray radiation was employed. The samples were analyzed under high-vacuum conditions in a multi-chamber UHV (ultra-high vacuum) system from Prevac, Poland. After mounting the samples on a molybdenum holder, they were degassed under steady high vacuum at room temperature in the load-lock chamber of the UHV system. Subsequently, the actual XPS analysis was conducted following the transfer of the holder with the sample to the analytical chamber of the UHV system. Monochromatic radiation with Al Kα characteristic line at 1486.7 eV energy was used as the excitation source, originating from an aluminum anode.

## 3. Results

### 3.1. Chemical Structure of Cellulose-Nitrate-Based Artificial Leather after Aging, Determined via the FTIR-ATR Method

FTIR measurements utilizing ATR reflectance attachments were conducted for three samples with varying compositions of the outer coating layer.

**Figure 5:**
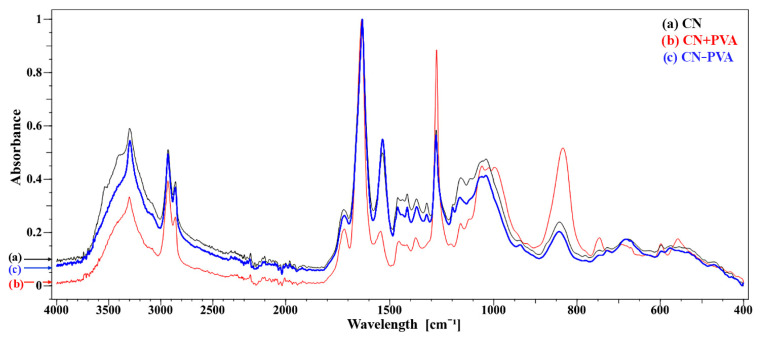
FTIR-ATR spectra for three samples subjected to artificial aging processes: (**a**) CN, (**b**) CN + PVA, and (**c**) CN − PVA.

A comparison of the spectra (Figure 5) indicates that the CN sample and the CN − PVA sample reacted similarly to the aging process.

The spectra obtained as a result of this study were compared with the FTIR spectrum of nitrocellulose included in the source material [35]. It was observed that the PVA-coated sample showed characteristic peaks corresponding to the nitro groups of nitrocellulose. The two intense and sharp bands at wavenumber values of approximately 1660 cm^−1^ and 1280 cm^−1^ come from the asymmetric stretching and symmetric stretching vibrations of the NO_2_ group, respectively. An equally intense band located at approximately 832 cm^−1^ is attributed to the stretching vibrations of the O-NO_2_ group. The low-intensity peak visible at approximately 748 cm^−1^ corresponds to asymmetric bending vibrations of O-NO_2_. The spectra obtained for the sample not covered with the PVA protective layer and for the sample with the PVA layer removed are characterized by reduced intensity of the mentioned bands characteristic of nitro groups of nitrocellulose.

By analyzing the spectra obtained during measurements using the FTIR-ATR technique, similar relationships were observed in terms of the position and intensity of the bands for the sample without the protective coating (CN) and the sample with the protective layer removed (CN–PVA). The latter sample was slightly more degraded, which is confirmed by the higher intensities of the valence vibration bands coming from the hydroxyl group located at the value of 3302 cm^−1^ resulting from the substitution of nitro groups of nitrocellulose with hydroxyl groups. Also, a slight decrease in the intensity of the O–NO_2_ stretching vibration bands at 1635 cm^−1^ and 1275 cm^−1^ indicates a greater degree of nitrocellulose degradation in this sample. Additionally, in the sample of the material from which the protective layer was removed, there was a visible increase in the intensity of the stretching bands of deformation vibrations δ(C–H) of aliphatic bonds at the positions 1462 and 1375 cm^−1^ and stretching ν(C–O) in the region of 1200–1000 cm^−1^.

The sample covered with a protective layer of poly(vinyl alcohol) (CN + PVA) had the lowest degree of degradation. The obtained FTIR-ATR spectrum is characterized by a much higher degree of cellulose nitration and, therefore, a lower degree of polymer degradation. This is evidenced by the already mentioned higher intensities of the spectral bands associated with valence vibrations located at 1635 and 1275 cm^−1^ and bending vibrations at 834 cm^−1^ coming from bonds in nitro groups. Additional confirmation is provided by the reduction in the intensity of the valence vibration band of –OH groups at 3303 cm^−1^ and the ν(C–O) vibration band in the region of 1200–1000 cm^−1^.

### 3.2. Elemental Composition of Cellulose-Nitrate-Based Artificial Leather after Aging, Determined via X-ray Photoelectron Spectroscopy (XPS)

**Figure 6:**
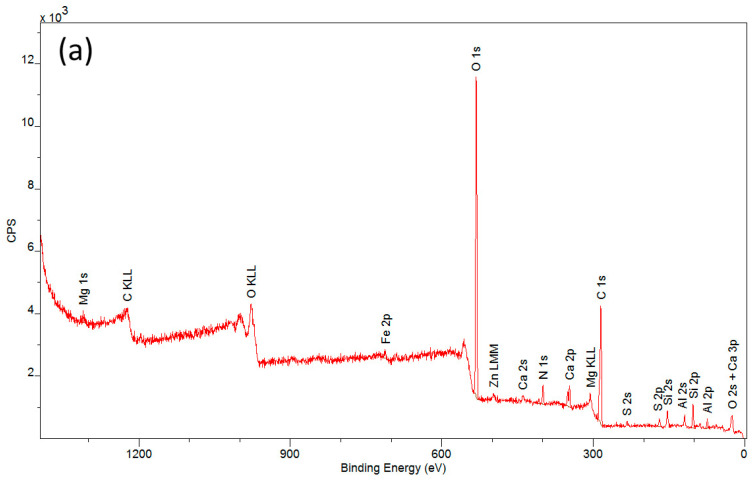

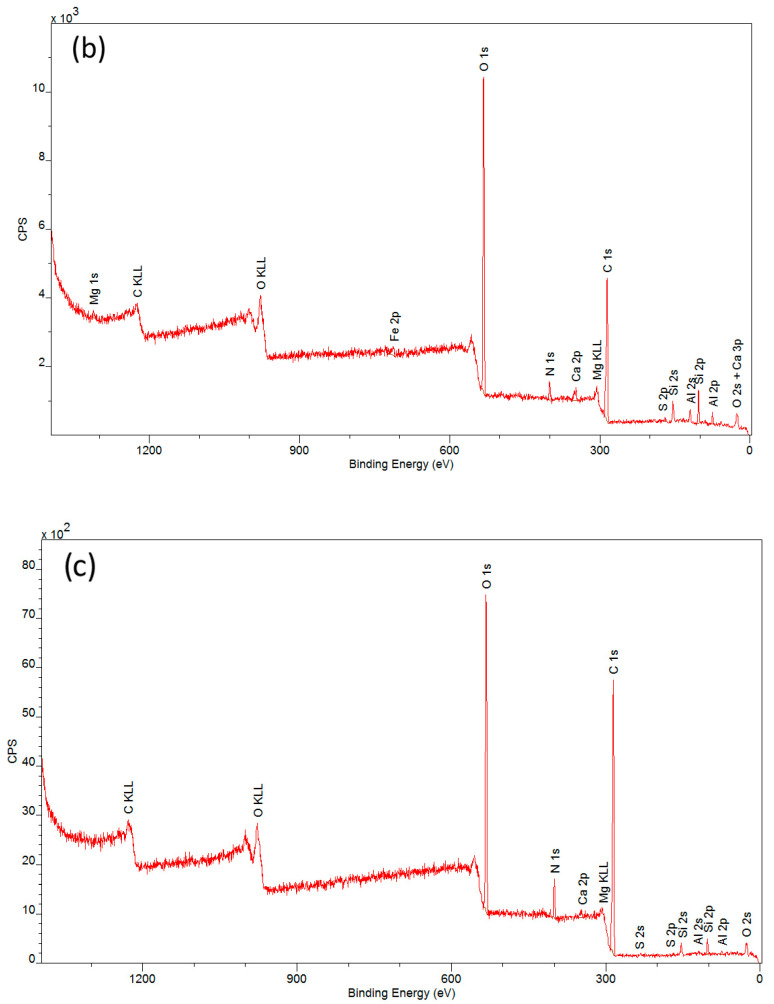
XPS spectra of samples: (**a**) an artificial leather sample without protective coating (CN), (**b**) an artificial leather sample with protective coating (CN + PVA), and (**c**) an artificial leather sample with the protective coating removed (CN − PVA).

A complementary technique used in studies concerning the applicability of a protective layer of polyvinyl alcohol is X-ray photoelectron spectroscopy (XPS). The spectra obtained from the measurements (Figure 6) allowed for the determination of the elemental composition of the outer coating layer covering the tested materials after the degradation process.

Detailed descriptions of the composition of the tested samples are provided in Table 1. Carbon and oxygen were predominant in all samples. Significant amounts of silicon and aluminum were also observed, along with other elements such as Mg, S, Ca, and Fe occurring in small quantities and varying proportions. These findings confirm the presence of a secondary layer on the surfaces of the investigated historical materials. The analyzed samples exhibited very low nitrogen content (ranging from 2.7% to 3.8%) in comparison to the maximum possible nitrogen content in cellulose nitrate, which is 11–12% [36]. The low nitrogen content is due to the decomposition of nitrocellulose to cellulose. This process occurs as a result of the aging of the historic material and involves the detachment of nitro groups and their replacement with hydroxyl groups. The elimination of nitro groups results in a reduction in the percentage of nitrogen in the analyzed samples.

**Figure 7:**
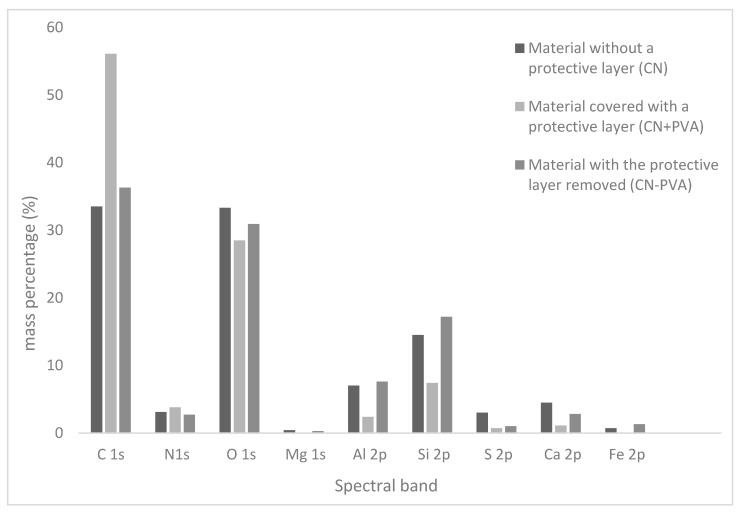
Percentage distribution of individual elements constituting the outer layer of the investigated cellulose-nitrate-based artificial leather samples.

Figure 7 depicts the percentage distribution (expressed as mass percentages) of individual elements constituting the outer layer of the investigated leather-like material samples.

The percentage content of nitrogen on the surface of the tested samples changed in the following order: CN − PVA < CN < CN + PVA (Table 2).

That the highest nitrogen content occurred in the material coated with a protective layer may suggest that polyvinyl alcohol to some extent protects the sample’s surface from cellulose nitrate degradation. The CN + PVA sample also exhibited the highest carbon content and the lowest oxygen content. This is attributed to the partial or complete removal of metal oxides and carbonates from the cellulose nitrate surface, as confirmed by the diagram in Figure 6. The N:C ratio on the surfaces of the examined materials increased in the order CN + PVA < CN − PVA < CN, and it was not directly correlated with the nitrogen content. Similarly irregular changes were observed in the degree of surface oxidation, expressed as the C:O ratio, where CN < CN − PVA << CN + PVA. According to this criterion, the lowest degree of oxidation characterizes the surface of the sample protected by the PVA film layer. The sample with the protective layer also exhibited the highest content of associated nitro groups O-NO_2_, amounting to 13.4% (%At). The respective quantities of associated nitro groups in the samples correlate with the nitrogen content determined for each material, indicating that the fewest associated nitro groups were registered for the sample with the protective layer removed.

All analyzed samples exhibited a low degree of substitution (DS): 0.4 for the sample without the protective layer, 0.5 for the sample coated with polyvinyl alcohol removed after the aging process, and 0.34 for the sample with the protective layer removed before degradation. Statistically, this means that one nitro group corresponds to at most half of a hydroxyl group. In practice, this indicates that the near-surface layer is almost entirely devoid of nitro groups.

## 4. Discussion

The collections at A-BSM contain numerous objects with components made entirely or partially of cellulose nitrate. Among them are suitcases covered with cellulose-nitrate-based artificial leather on the exterior. One of the conservation challenges pertains to the secondary protective coating of PVA applied several decades ago onto the surfaces of historic objects, including artificial leather. The secondary coating was intended to protect the original material from degradation. The purpose of conducting these studies was to facilitate the decision-making process regarding the removal or retention of the PVA layer on the suitcases, as well as to address whether removing the PVA could accelerate the degradation process.

Aqueous PVA solutions have been used for the preservation of cultural heritage objects since the mid-20th century. They have been applied as protective coatings or consolidants for paint layers. PVA has also found utility as an adhesive for conserving paper, parchment, and textiles, and even for reinforcing paleontological objects [37,38,39]. In A-BSM, it was used for protecting fabrics, paper, and artificial leather.

Research making use of the compatibility of cellulose and PVA has been conducted for several decades. These studies aimed at obtaining composites from these two polymers [40,41,42,43,44]. Both polymers are polar, hydrophilic, and biocompatible, and researchers expected to obtain stable composites with good mechanical properties. The studies revealed enhanced molecular interactions between both polymers, primarily formed through hydrogen bonding between cellulose and PVA molecules. The authors demonstrated that the resulting composites exhibit good mechanical, barrier, and antimicrobial properties.

However, scientific research on protective PVA coatings used in conservation has shown that these coatings are not durable and undergo rapid physicochemical changes [45,46].

Studies on cellulose-nitrate-based coatings on artificial leather have not been explored in previously published scientific research. The focus of conservators and researchers studying the composition and degradation process of cellulose nitrate in cultural heritage objects has primarily centered on motion picture films [16,23,33,47,48], as well as three-dimensional heritage objects entirely constructed from cellulose nitrate [28,47,49,50,51], wood lacquers [13], and cellulose nitrate as a conservation agent [27,52].

These studies have revealed the complexity of cellulose nitrate degradation processes. Chemical, thermal, photochemical, and physical causes of these processes were investigated. Loss of camphor, the breakdown of the polymer’s main chain, and denitration leading to the formation of nitrogen oxides and related products, as well as the autoreactive nature of these processes, were described. The importance of sulfate residues from the stabilization process in CN production was also highlighted.

The unstable artifacts made from cellulose nitrate stored in museum collections prompted researchers to examine the degradation processes of this material. In order for these processes to be observed within relatively short research periods compared to natural degradation processes, accelerated aging of samples is employed. Various conditions for the accelerated aging of cellulose nitrate have been proposed, deviating more or less from the conditions in museum storage. Some of these conditions included 60 °C, 70 °C, and 100 °C with UV–vis exposure [27]; 70 °C and 75% RH [28]; 60 °C and 75% RH [34]; and 60 °C with UV–vis exposure [29]. Comparative studies of the degradation rates of cellulose nitrate under different climatic conditions have also been conducted, such as under 12%, 15%, and 75% RH at 70 °C [15], as well as 30%, 50%, and 80% RH at 60 °C [33]. These comparisons have shown that degradation processes actively occur at lower temperatures and humidities, although at a slower pace than under higher-parameter conditions, and they can be documented using analytical methods. Citing previous research results, the conditions chosen for this study, including 40% RH, 50 °C, and UV–vis exposure, were deemed sufficient to highlight differences in the degradation rates of samples without inducing changes in the PVA coating.

Published results of XPS studies applied to cellulose nitrate in historical artifacts are not available. Cellulose nitrate has been studied using this technique in industry, confirming its usefulness in investigating the degradation mechanism of cellulose nitrate [31,32]. This technique has also been used to understand the degradation processes of PVA used in the conservation of paint layers [46].

The results in this study obtained through XPS spectroscopy largely align with the data obtained from FTIR-ATR measurements. In both conducted studies, the sample of material covered with the protective layer of polyvinyl alcohol (CN + PVA) exhibited the least degradation, associated with the highest content of nitro groups on the surface of the analyzed material. In the case of the other two samples, the results are not unequivocally correlated. The FTIR-ATR spectrum obtained for the sample with the protective layer removed (CN − PVA) indicated the highest degree of degradation compared to the other samples. However, the data obtained from XPS measurements suggest that it is not possible to definitively determine which sample, CN or CN − PVA, underwent a higher degree of aging.

A limitation of the methodology used in this study is the inability to obtain samples from historical artifacts where the protective PVA coating was applied several decades ago and aged alongside the original material. Another challenge arises from the fact that artificial leather produced in different factories or even different countries, based on various patents and formulations, may differ in its components and proportions. The different composition of the coating material could lead to distinct degradation processes.

In this study, the focus was solely on one type of material used in conservation as a secondary protective layer. Exploring the impact of other membrane-forming materials seems advisable and intriguing.

## 5. Conclusions

The aim of this study was to determine whether the protective PVA coating applied several decades ago to artificial leather prevented its deterioration or accelerated its degradation. This research also aimed to decide whether PVA should be removed during the conservation of suitcases in A-BSM and whether this action is safe for the original coating. The results obtained through FTIR-ATR and XPS techniques indicated the protective effect of the PVA layer in the aging process of cellulose nitrate. Both methods confirmed that in the sample protected with a PVA coating, the degradation processes occurred to the least extent. However, they did not provide a definitive conclusion on whether water-based removal of PVA had a destructive impact on the original coating or had no effect.

## Figures and Tables

**Table 1:** Elemental surface composition analysis of the samples CN, CN + PVA, and CN − PVA.

Spectral Band	CN			CN + PVA			CN − PVA		
	E_B_/eV	% At	% Mass	E_B_/eV	% At	% Mass	E_B_/eV	% At	% Mass
C 1s	284.99	45.7	33.5	285.00	65.5	56.1	284.98	48.9	36.3
N 1s	399.49	3.7	3.1	399.50	3.8	3.8	399.98	3.1	2.7
O 1s	531.99	34.1	33.3	532.50	25.0	28.5	531.98	31.3	30.9
Mg 1s	1310.49	0.3	0.4	-	-	-	1311.48	0.2	0.3
Al 2p	73.99	4.3	7.0	75.00	1.3	2.4	74.48	4.5	7.6
Si 2p	102.49	8.5	14.5	102.00	3.7	7.4	102.98	10.0	17.2
S 2p	168.49	1.5	3.0	168.50	0.3	0.7	169.48	0.5	1.0
Ca 2p	346.99	1.9	4.5	348.00	0.4	1.1	347.48	1.1	2.8
Fe 2p	711.99	0.2	0.7	-	-	-	713.48	0.4	1.3

**Table 2:** Percentage content and ratio of elements/groups on the surface of the tested samples.

Element/Group	CN	CN + PVA	CN − PVA
N [%]	3.10	3.80	2.70
C [%]	33.50	56.10	36.30
O [%]	33.30	28.50	30.90
N:C	1:11	1:15	1:13
C:O	1:1	4.6:1	1.2:1
O-NO_2_ [%At]	10.7	13.4	8.0
DS	0.40	0.50	0.34

## Data Availability

No new data has been created.
